# Supplementary material for: Mesophotic coral bleaching associated with changes in thermocline depth
Source: Nat Commun. 2023 Oct 16;14:6528. doi: 10.1038/s41467-023-42279-2 (PMC10579316; doi:10.1038/s41467-023-42279-2)
Supplement: Supplementary file 1 — Supplementary Information [file 41467_2023_42279_MOESM1_ESM.pdf]

Supplementary information

Mesophotic coral bleaching associated with changes in thermocline depth

MS CLARA DIAZ[1†*], DR NICOLA L. FOSTER[1†*], PROF MARTIN J. ATTRILL[1], MR ADAM BOLTON[1], MR PETER GANDERTON[1], PROF KERRY L. HOWELL[1], MR EDWARD ROBINSON[1], DR PHILIP HOSEGOOD[1†*]

*These authors contributed equally to this work.
† corresponding authors

[1]School of Biological and Marine Sciences, University of Plymouth, Drake Circus, Plymouth, PL4 8AA, United Kingdom.

Table S1: Total colonies and species numbers of photosynthetic scleractinian corals recorded per season, site and depth band. NOV = November 2019; MAR = March 2020; IDR = Ile Des Rats; MA = Manta Alley

Season	Site	Depth band	Image number	Colonies number	Species number
NOV	IDR	15-20m	90	2254	93
NOV	IDR	30-40m	90	535	51
NOV	IDR	60-70m	90	248	15
NOV	IDR	80-90m	90	420	2
NOV	MA	15-20m	60	513	55
NOV	MA	60-70m	60	79	9
NOV	MA	80-90m	60	142	2
MAR	MA	15-20m	30	1005	86
MAR	MA	30-40m	60	178	50
MAR	MA	60-70m	30	270	12
MAR	MA	80-90m	30	34	2

Table S2-1: two-sided Permutational Analysis of variance (PERMANOVA) test results with presence/absence data and Bray-Curtis similarity. 999 permutations. Pairwise tests between the two studied sites, Ile Des Rats (IDR) and Manta Alley (MA) for each depth band at Egmont Atoll. P(perm): p-value

Groups observed	T test	P (perm)	Unique permutations
IDR 80 – 90m, IDR 60 – 70m	3.6111	0.001	999
IDR 80 – 90m, IDR 30 – 40m	8.7005	0.001	998
IDR 80 – 90m, IDR 15 – 20m	15.109	0.001	998
IDR 60 – 70m, IDR 30 – 40m	6.9386	0.001	997
IDR 60 – 70m, IDR 15 – 20m	13.367	0.001	997
IDR 30 – 40m, IDR 15 – 20m	7.1279	0.001	999
MA 80 – 90m, MA 60 – 70m	4.1598	0.001	999
MA 80 – 90m, MA 30 – 40m	4.9473	0.001	998
MA 80 – 90m, MA 15 – 20m	12.379	0.001	996
MA 60 – 70m, MA 30 – 40m	6.2196	0.001	999
MA 60 – 70m, MA 15 – 20m	10.741	0.001	999
MA 30 – 40m, MA 15 – 20m	9.0945	0.001	999
IDR 80 – 90m, MA 80 – 90m	5.4118	0.001	999
IDR 60 – 70m, MA 60 – 70m	1.958	0.022	997
IDR 30 – 40m, MA 30 – 40m	5.4893	0.001	998
IDR 15 – 20m, MA 15 – 20m	2.5895	0.001	998

Table S2-2: Average similarity between/within groups of two-sided PERMANOVA test, with abundance data, square-root transformed, and Bray-Curtis similarity, 999 permutations. Pairwise tests between the two studied sites, Ile Des Rats (IDR) and Manta Alley (MA) for each depth band at Egmont Atoll.

	IDR-15	IDR-30	IDR-60	IDR-80	MA-15	MA-30	MA-60	MA-80
**IDR-15**	45.554	26.885	12.562	12.04	40.199	18.718	12.008	12.871
**IDR-30**		38.68	34.164	31.652	28.687	36.74	31.251	36.585
**IDR-60**			61.27	61.594	16.764	51.945	57.529	66.351
**IDR-80**				69.717	15.909	46.195	59.776	65.346
**MA-15**					38.735	22.559	15.851	17.569
**MA-30**						55.805	45.356	58.648
**MA-60**							55.767	60.134
**MA-80**								73.737

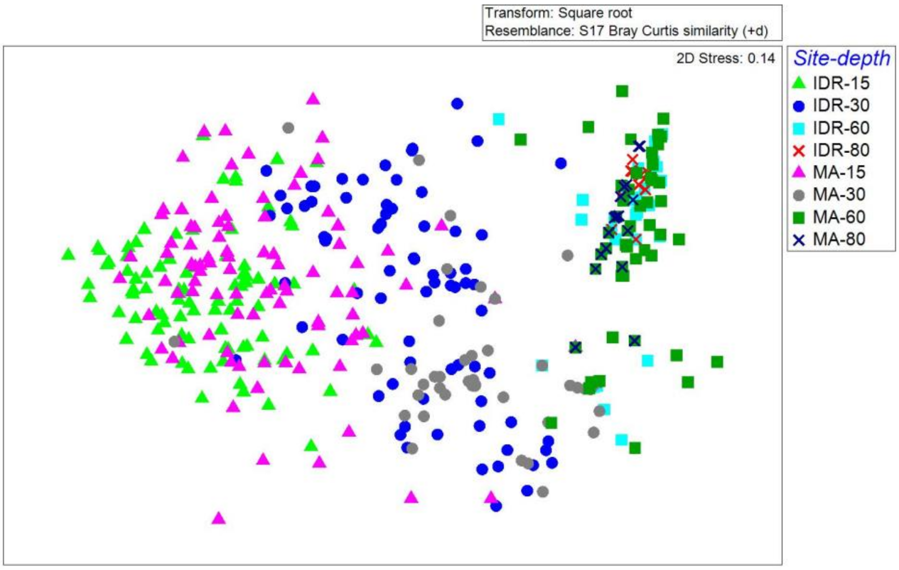

Figure S1: Multidimensional scaling (MDS) plot on scleractinian coral abundance, square-root transformed and balanced data, with Bray Curtis similarity. A dummy variable was added to account for zero inflated data. IDR: Ile Des Rats; MA: Manta Alley. Depth in m.

Table S3: Number of shared photosynthetic scleractinian coral species between season, site and depth band. N: November 2019; M: March 2020; IDR: Ile Des Rats; MA: Manta Alley; 15: 15-20m; 30: 30-40m; 60: 60-70m; 80: 80-90m

	**N-IDR-30**	**N-IDR-60**	**N-IDR-80**	**N-MA-15**	**N-MA-60**	**N-MA-80**	**M-MA-15**	**M-MA-30**	**M-MA-60**	**M-MA-80**
**N-IDR-15**	28	5	0	42	2	0	60	35	2	0
**N-IDR-30**		10	2	20	6	2	28	26	8	2
**N-IDR-60**			2	3	8	2	5	6	8	2
**N-IDR-80**				0	2	2	0	1	2	2
**N-MA-15**					3	0	40	25	3	0
**N-MA-60**						2	2	3	8	2
**N-MA-80**							0	1	2	2
**M-MA-15**								37	1	0
**M-MA-30**									3	1
**M-MA-60**										2

Table S4: Bleaching Index (BI) and prevalence (%) values at Ile des Rats (IDR) and Manta Alley in November 2019, for each species observed throughout a large depth range (15-20m m deep to 60-70 m and down to 80-90 m for some species). x: no data. N = total number of individuals.

			Depth Bands						
Sites	Scleractinian species	Bleaching	15-20 m		30 – 40 m		60 – 70 m		80 – 90 m
	IOTU49	BI	1		1.66		0		X
	Pachyseris sp.			*N=40*		*N = 85*	0	*N = 9*	
	N = 134	%	2.5		3.53		0		X
IDR	*IOTU48*	BI	2.33		2.66		1		X
	Leptoseris sp.			*N=25*		*N = 43*		*N = 2*	
	N = 70	%	28		18.60		50		X
		BI	0.33	*N=8*	0	*N = 3*	0	*N = 1*	X

	IOTU534 Leptoseris sp. N = 12	%	12.5		0		0		X	
	IOTU47 Oxypora sp. N = 11	BI	0	*N=1*	0	*N = 8*	0	*N = 2*	X	
		%	0		0		0		X	
	IOTU44 Leptoseris sp. N = 234	BI	7.66	*N= 154*	7.33	*N = 78*	0	*N = 2*	X	
		%	12.99		23.08		0		X	
	IOTU45 Leptoseris sp. N = 3	BI	X		0	*N = 2*	0.33	*N = 1*	X	
		%	X		0		100		X	
	IOTU529 Leptoseris sp. N = 10	BI	X		0	*N = 1*	0	*N = 9*	X	
		%	X		0		0		X	
	IOTU533 Leptoseris sp. N = 6	BI	X		1.66	*N = 5*	0.33	*N = 1*	X	
		%	X		60		100		X	
	IOTU340 Leptoseris sp. N = 566	BI	X		0.33	*N = 14*	78.33	*N = 162*	171.33	*N = 390*
		%	X		7.14		86.42		81.21	
	IOTU416 Leptoseris sp. N = 76	BI	X		3.33	*N=5*	32.33	*N = 52*	11	*N = 19*
		%	X		80		88.46		84.21	
MA	*IOTU48 Leptoseris sp. N = 15*	BI	1.66	*N= 14*	X		1	*N = 1*	X	
		%	35.71		X		100		X	
	IOTU44 Leptoseris sp. N = 67	BI	7	*N=66*	X		0	*N=1*	X	
		%	30.30		X		0		X	
	IOTU418 *Leptoseris sp. N= 3*	BI	0	*N=1*	X		0	*N=2*	X	
		%	0		X		0		X	
	IOTU340 *Leptoseris sp. N=70*	BI	X		X		28.66	*N=52*	11.66	*N=139*
		%	X		X		80.77		77.77	

IOTU416	BI	X	X	101		2.33	
Leptoseris sp.					*N=18*		*N=3*
N=142	%	X	X	98.56		100	

Table S5: Bleaching Index (BI) detailed results at Ile des Rats (IDR) and Manta Alley in November 2019 and March 2020. BI p-values for November 2019 by depth & site: Kruskal-Wallis chi-squared = 2393.1, df = 6, p-value < 2.2e-16. BI p-values for March 2020 by depth & site: Kruskal-Wallis chi-squared = 804.64, df = 6, p-value < 2.2e-16. Details of pairwise comparisons in November (N) and March (M) between sites and depth bands (15: 15-20m; 30: 30-40m; 60: 60-70m; 80: 80-90m); using Wilcoxon rank sum test with continuity correction and Benjamini-Hochberg (BH) adjusted method. Non-significant data are shown in bold.

	**N-IDR-30**	**N-IDR-60**	**N-IDR-80**	**N-MA-15**	**N-MA-60**	**N-MA-80**	**M-MA-15**	**M-MA-30**	**M-MA-60**	**M-MA-80**
**N-IDR-15**	1.5e-05	< 2.2e-16	< 2.2e-16	1.9e-07	< 2.2e-16	< 2.2e-16	-	-	-	-
**N-IDR-30**		< 2.2e-16	< 2.2e-16	**0.58**	< 2.2e-16	< 2.2e-16	-	-	-	-
**N-IDR-60**			**0.51**	< 2.2e-16	**0.86**	6.0e-08	-	-	-	-
**N-IDR-80**				< 2.2e-16	**0.79**	3.1e-07	-	-	-	-
**N-MA-15**					< 2.2e-16	< 2.2e-16	**0.63852**	**0.28911**	< 2.2e-16	0.00062
**N-MA-60**						8.3e-07	< 2.2e-16	< 2.2e-16	4.2e-05	1.0e-06
**N-MA-80**							< 2.2e-16	< 2.2e-16	< 2.2e-16	< 2.2e-16
**M-MA-15**								**0.13989**	< 2.2e-16	0.00014
**M-MA-30**									< 2.2e-16	0.01720
**M-MA-60**										0.01440

Table S6: Bleaching Prevalence detailed results at Ile des Rats (IDR) and Manta Alley in November 2019 and March 2020. BI p-values for November 2019 by depth & site: Kruskal-Wallis chi-squared = 2422.5, df = 6, p-value < 2.2e-16. BI p-values for March 2020 by depth & site: Kruskal-Wallis chi-squared = 883.55, df = 6, p-value < 2.2e-16. Details of pairwise comparisons in November (N) and March (M) between sites and depth bands (15: 15-20m; 30: 30-40m; 60: 60-70m; 80: 80-90m); using Wilcoxon rank sum test with continuity correction and Benjamini-Hochberg (BH) adjusted method. Non-significant data are shown in bold.

	N-IDR-30	N-IDR-60	N-IDR-80	N-MA-15	N-MA-60	N-MA-80	M-MA-15	M-MA-30	M-MA-60	M-MA-80
**N-IDR-15**	1.3e-05	< 2.2e-16	< 2.2e-16	2.1e-07	< 2.2e-16	< 2.2e-16	-	-	-	-
**N-IDR-30**		< 2.2e-16	< 2.2e-16	**0.574**	< 2.2e-16	< 2.2e-16	-	-	-	-
**N-IDR-60**			**0.340**	< 2.2e-16	**0.075**	1.9e-05	-	-	-	-
**N-IDR-80**				< 2.2e-16	**0.105**	4.0e-12	-	-	-	-
**N-MA-15**					< 2.2e-16	< 2.2e-16	**0.72155**	**0.29527**	< 2.2e-16	0.00089
**N-MA-60**						1.4e-06	< 2.2e-16	< 2.2e-16	4.7e-11	8.3e-08
**N-MA-80**							< 2.2e-16	< 2.2e-16	0.00024	8.3e-09
**M-MA-15**								**0.16807**	< 2.2e-16	0.00027
**M-MA-30**									< 2.2e-16	0.02156
**M-MA-60**										0.01061

Table S7: 10%, 1% and 0.1% light optical depths for each deployment. Depths are expressed in metres, with downward and upward light optical depths measured for each deployment undertaken in 2020 and 2022. Dates are expressed as month/year, each date is a different day.

**Date**	03/20	03/20	03/20	03/20	03/20	03/20	03/22	03/22	03/22	03/22	03/22
**10% light optical depth (m) = 2.3/K_D downward & upward**	48.9<br>37.7	41.1<br>43.4	38.3<br>34.3	31.5<br>36.5	41.8<br>65.7	45.1<br>56.1	41.8<br>40.3	43.4<br>44.2	40.3<br>43.4	47.9<br>50	46<br>52.3
**1% light optical depth (m) = 4.6/K_D downward & upward**	97.8<br>75.4	82.2<br>86.8	76.6<br>68.6	63<br>73	83.6<br>131.4	90.2<br>112.2	83.6<br>80.6	86.8<br>88.4	80.6<br>86.8	95.8<br>100	92<br>104.6
**0.1% light optical depth (m) = 6.8/K_D downward & upward**	144.7<br>111.5	121.4<br>128.3	113.3<br>101.5	93.2<br>107.9	123.6<br>194.3	133.3<br>165.8	123.6<br>119.3	128.3<br>130.8	119.3<br>128.3	141.7<br>147.8	136<br>154.5

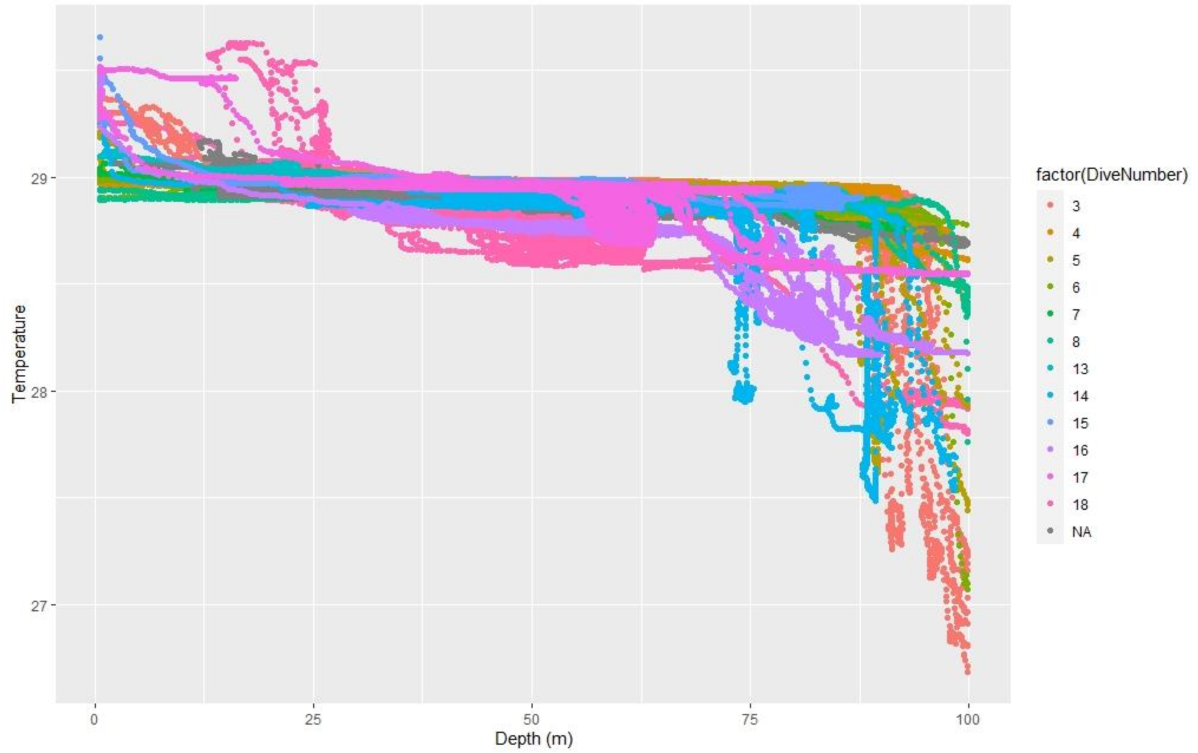

Figure S2: Temperature with depth of the different ROV dives. Temperature was measured via a CTD mounted on the ROV. The different colours show the different ROV dives undertaken in November 2019.

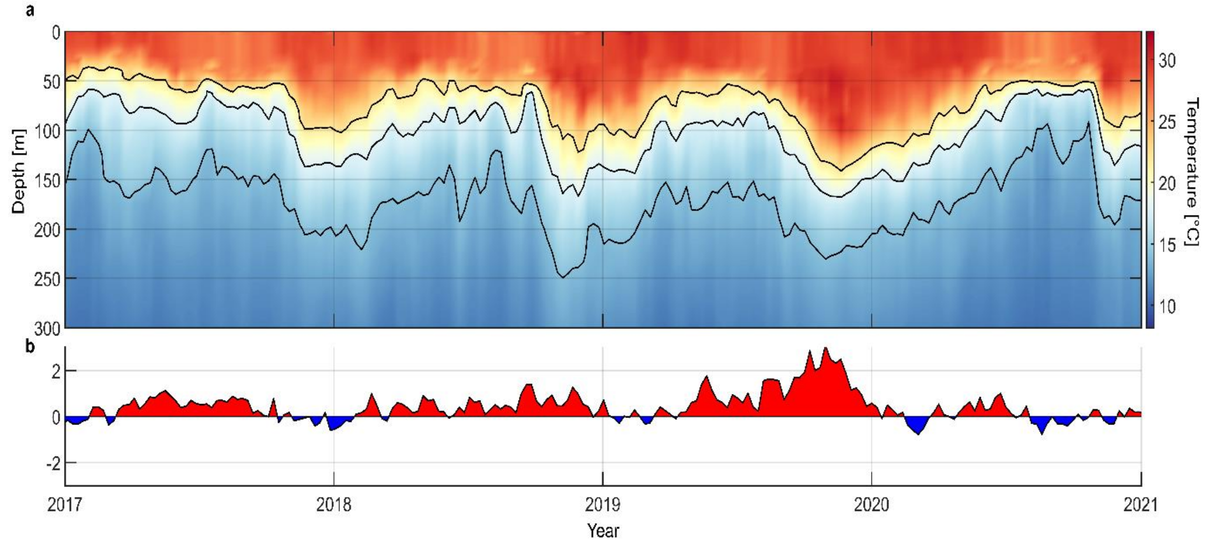

Figure S3: Local temperature fluctuation in the Chagos Archipelago a) Temperature (°C) of the upper 300m between 2017 and 2021 in the Chagos Archipelago. The three isobaths indicate 22°C, 18°C and 14°C. b) corresponding IOD index.

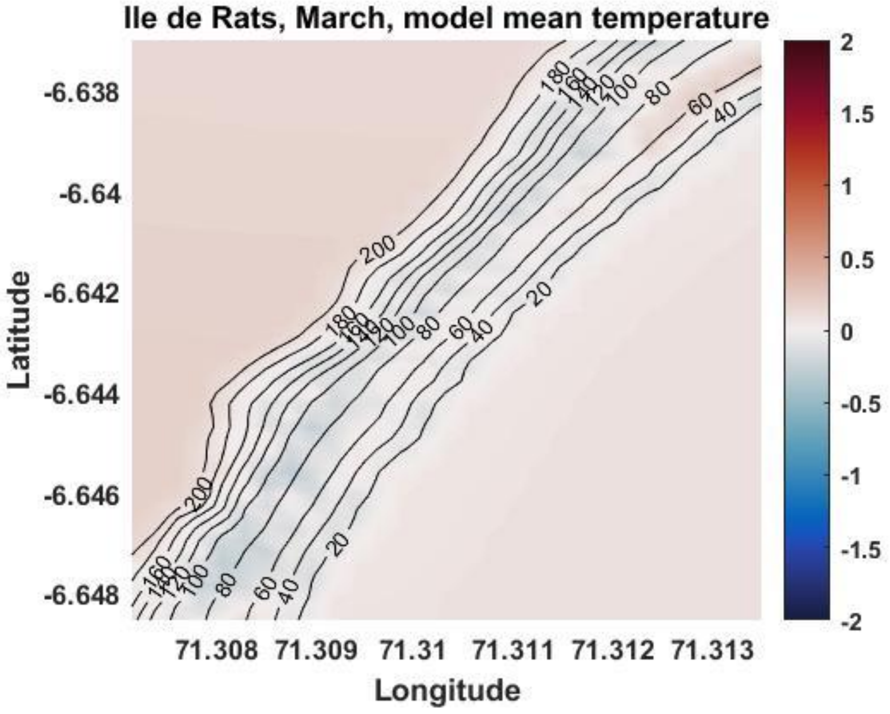

Figure S4: Model-predicted bottom temperature variations at Ile Des Rats, March 2020. The isobaths (solid lines with the labels in meters) at Ile des Rats are overlayed with the model-predicted bottom temperature variations calculated over a 15-day cycle.

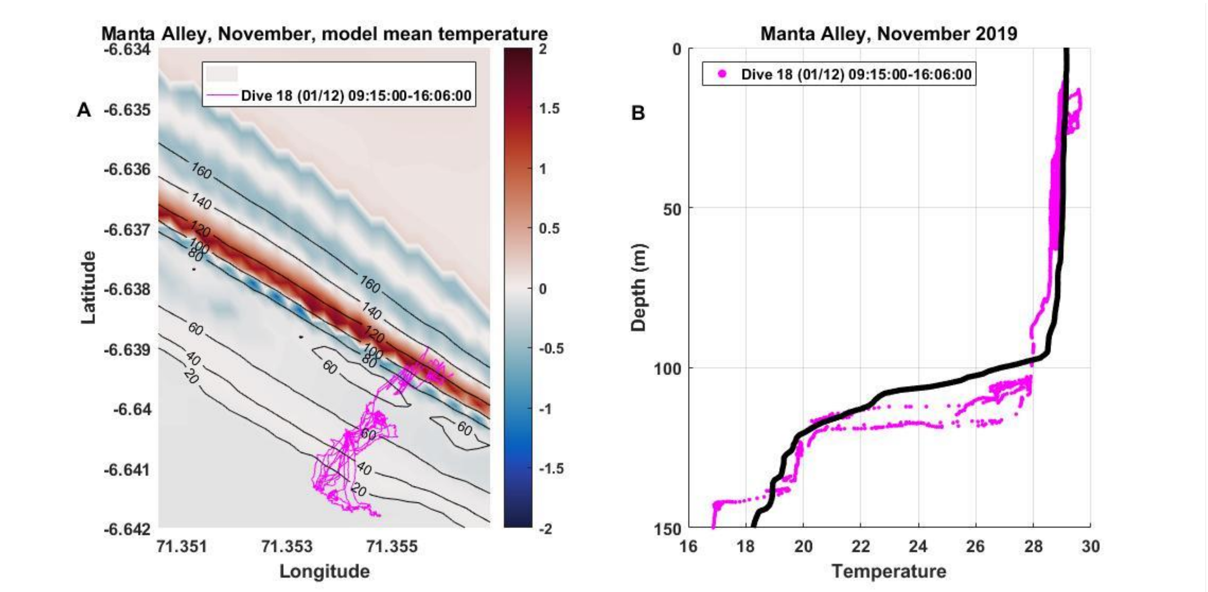

Figure S5: A) Model-predicted bottom temperature variations at Manta Alley, November 2019, calculated over a 15-days cycle. The temperature panel is overlaid with the isobaths (solid lines with labels). The track of the ROV dive is shown by magenta. B) In-situ water temperature recorded with the ROV (shown in panel A) in Manta Alley. The solid black line in panel B shows the background temperature profile recorded in November 2019.

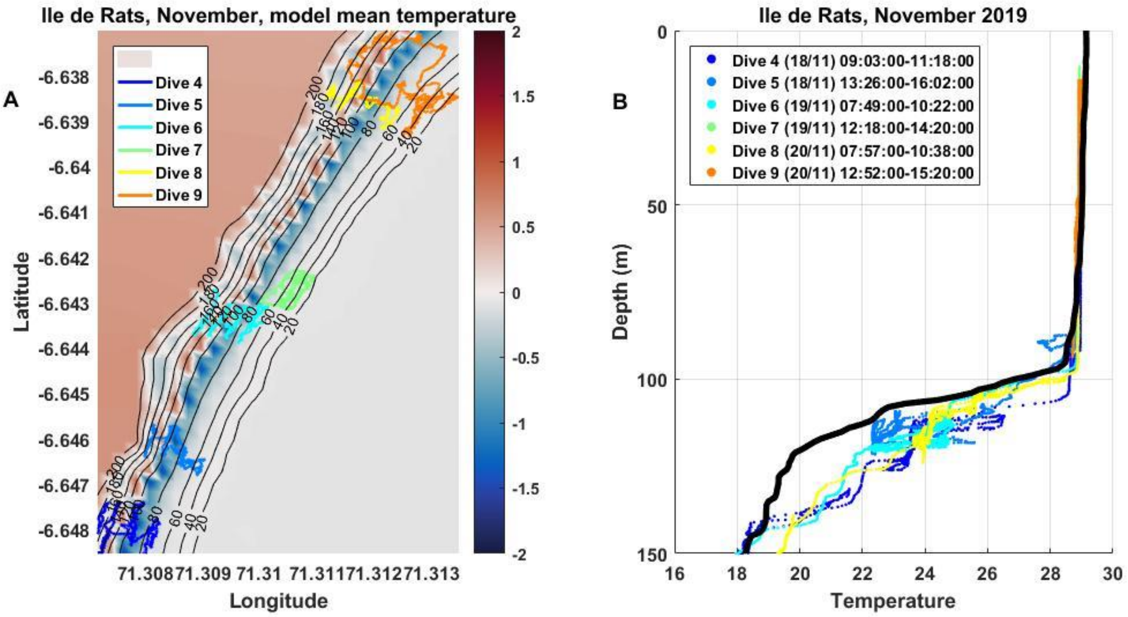

Figure S6: A) Model predicted bottom temperature variations at Ile Des Rats, November 2019, calculated over a 15-days cycle. The temperature panel is overlaid with isobaths (solid lines with the labels in meters). B) In-situ water temperature recorded with the ROV (shown in panel A) in Manta Alley. The colours of the temperature records in panels A and B coincide. The solid black line in panel B shows the background temperature profile recorded in November 2019.

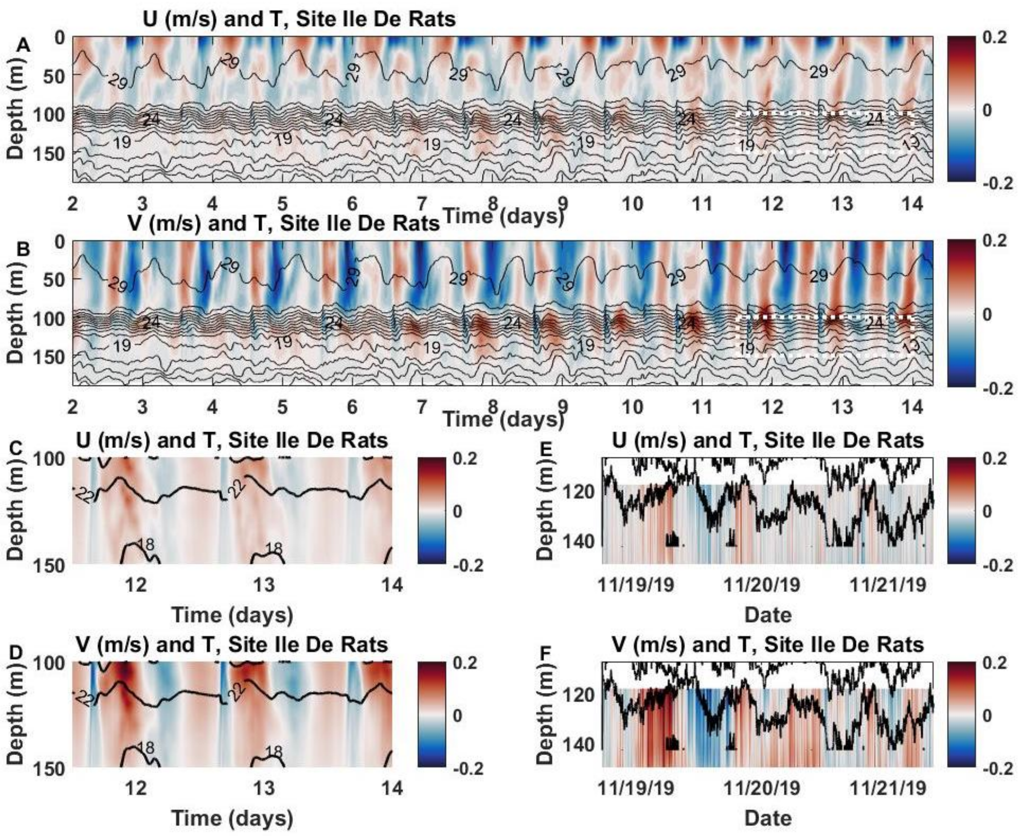

Figure S7: Predicted velocity time series overlaid with temperature isolines at Ile Des Rats, November 2019, for A) zonal and B) meridional. The position of the ADCP and thermistor chain are shown in Figure 8a. Data in the white rectangle C) and D) are used for comparison with similar in-situ data E) and F).

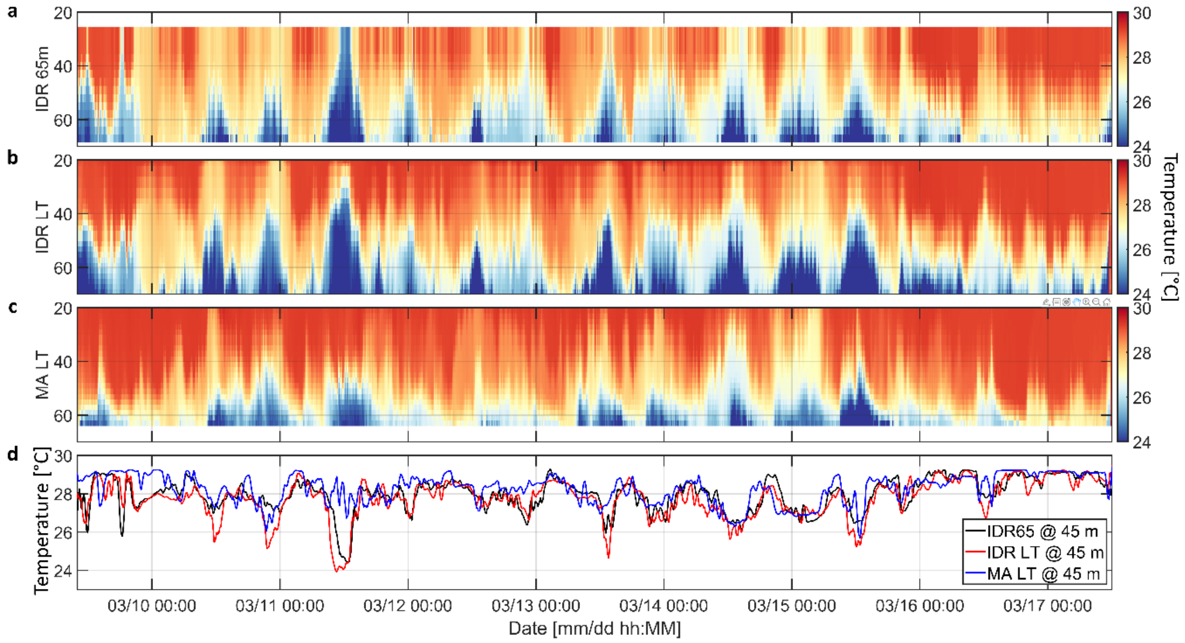

Figure S8: Temperature over depth and time during March 2020 when three moorings simultaneously monitored the temperature and currents in water of 65 m depth at a) west Ile Des Rats (IDR), b) east IDR and c) Manta Alley (MA); the instantaneous temperature at 45 m depth at each mooring, d), illustrates the significantly stronger cooling effect of the waves at both IDR sites compared to MA, whereby the waves were of larger amplitude and extended over a greater vertical range than in MA.
