# Supplementary material for: Mesophotic coral bleaching associated with changes in thermocline depth
Source: Nat Commun. 2023 Oct 16;14:6528. doi: 10.1038/s41467-023-42279-2 (PMC10579316; doi:10.1038/s41467-023-42279-2)
Supplement: Supplementary file 3 — Reporting Summary [file 41467_2023_42279_MOESM3_ESM.pdf]

nature portfolio

Corresponding author(s): Clara Diaz, Nicola L. Foster, Phil Hosegood

Last updated by author(s): 14/09/2023

Reporting Summary

Nature Portfolio wishes to improve the reproducibility of the work that we publish. This form provides structure for consistency and transparency in reporting. For further information on Nature Portfolio policies, see our Editorial Policies and the Editorial Policy Checklist.

Statistics

For all statistical analyses, confirm that the following items are present in the figure legend, table legend, main text, or Methods section.

n/a	Confirmed	
☒	☐	The exact sample size (*n*) for each experimental group/condition, given as a discrete number and unit of measurement
☒	☐	A statement on whether measurements were taken from distinct samples or whether the same sample was measured repeatedly
☐	☒	The statistical test(s) used AND whether they are one- or two-sided<br>*Only common tests should be described solely by name; describe more complex techniques in the Methods section.*
☒	☐	A description of all covariates tested
☐	☒	A description of any assumptions or corrections, such as tests of normality and adjustment for multiple comparisons
☒	☐	A full description of the statistical parameters including central tendency (e.g. means) or other basic estimates (e.g. regression coefficient) AND variation (e.g. standard deviation) or associated estimates of uncertainty (e.g. confidence intervals)
☐	☒	For null hypothesis testing, the test statistic (e.g. F, t, r) with confidence intervals, effect sizes, degrees of freedom and P value noted<br>*Give P values as exact values whenever suitable.*
☒	☐	For Bayesian analysis, information on the choice of priors and Markov chain Monte Carlo settings
☒	☐	For hierarchical and complex designs, identification of the appropriate level for tests and full reporting of outcomes
☒	☐	Estimates of effect sizes (e.g. Cohen's d, Pearson's r), indicating how they were calculated

Our web collection on statistics for biologists contains articles on many of the points above.

Software and code

Policy information about availability of computer code

Data collection	BIIGLE open source annotation software was used to annotate the images: https://biigle.de/. Oceanographic data were collected using the manufacturer's proprietary software; Nortek Signature Deployment, AquaPro (https://www.nortekgroup.com/software), Ocean Contour (not open source); RBR Ruskin (https://updates.rbr-global.com/software/ruskin/installer/RBRRuskin.dmg); Seastar (https://www.star-oddi.com/media/1/seastar.installer.offline-9.05.zip)
Data analysis	RStudio version 4.0.4; Primer v.6; Oceanographic and numerical modelling data were analysed with Matlab 2021b; code was written by Edward Robinson and Nataliya Stashchuk

For manuscripts utilizing custom algorithms or software that are central to the research but not yet described in published literature, software must be made available to editors and reviewers. We strongly encourage code deposition in a community repository (e.g. GitHub). See the Nature Portfolio guidelines for submitting code & software for further information.

Data

Policy information about availability of data

All manuscripts must include a data availability statement. This statement should provide the following information, where applicable:

- Accession codes, unique identifiers, or web links for publicly available datasets
- A description of any restrictions on data availability
- For clinical datasets or third party data, please ensure that the statement adheres to our policy

the morphospecies catalogue is currently published on zenodo: https://doi.org/10.5281/zenodo.7948517 and will be fully available on smartarID:https://smartar-id.app/. Some modelling data are available here: https://figshare.com/articles/dataset/Egmont2020/19925381. Data may be shared upon request but the authors reserve the right to retain ownership of the data for a reasonable period of time to enable the project researchers sufficient time to publish their findings.

Research involving human participants, their data, or biological material

Policy information about studies with human participants or human data. See also policy information about sex, gender (identity/presentation), and sexual orientation and race, ethnicity and racism.

Reporting on sex and gender	NA
Reporting on race, ethnicity, or other socially relevant groupings	NA
Population characteristics	NA
Recruitment	NA
Ethics oversight	NA

Note that full information on the approval of the study protocol must also be provided in the manuscript.

Field-specific reporting

Please select the one below that is the best fit for your research. If you are not sure, read the appropriate sections before making your selection.

☐ Life sciences ☐ Behavioural & social sciences ☒ Ecological, evolutionary & environmental sciences

For a reference copy of the document with all sections, see nature.com/documents/nr-reporting-summary-flat.pdf

Ecological, evolutionary & environmental sciences study design

All studies must disclose on these points even when the disclosure is negative.

Study description	This study was multidisciplinary, which includes biological, oceanographic and modelling data. It was about mesophotic coral ecosystems bleaching that occurred in the Chagos archipelago and more specifically at Egmont Atoll, in November 2019.
Research sample	90 images were collected at each depth (from 15m to 90m) for each two study sites of Egmont atoll, every scleractinian corals in the images were taken into account
Sampling strategy	No sample size was chosen, every scleractinian coral specimen observed were taken into account. Images were collected using a remotely operated vehicle. The sampling procedure can be consulted in the methods section of the main manuscript
Data collection	We undertook 2 research cruises where we collected the data: one in November 2019 and one in March 2020. within the atoll, 2 sites were surveyed: Ile Des Rats and Manta Alley. Within these two sites, 4 depth bands were targeted (15-20m; 30-40m; 60-70m; 80-90m) with a Remotely Operated Vehicle (ROV). Videos transects were conducted with the ROV and 30 images per transect were extracted. In November 2019, 3 transects were conducted at Ile des Rats and one in Manta Alley. In March 2020, 2 transects were conducted in Manta Alley only (we had to stop the research cruise earlier due to Covid-19 outbreak). Oceanographic data were collected during research cruises to the Indian Ocean aboard the Tethys Supporter. Data were obtained from subsurface taut line moorings that were deployed in the standard manner. Temperature data were obtained using RBR Solo T temperature sensors, CTD data were obtained using RBR Maestro CTDs, and ADCP data were acquired using a Nortek Signature 500 kHz ADCP and Nortek Aquadopp 400 kHz ADCP
Timing and spatial scale	Data were collected during two research cruises; the first one was from the 11th of November to the 7th of December 2019; the second one from the 5th to the 22nd of March 2020. These periods were chosen for logistical purposes. Image data were collected within days, while oceanographic data where collected consistently between November and March.
Data exclusions	no data were excluded from the analysis

Reproducibility	This study investigates a rare event, so cannot be reproducible
Randomization	allocation was not random, samples were placed into groups based on depth, site and level of bleaching
Blinding	blinding was not relevant in this study because we were studying a natural phenomenon in the field

Did the study involve field work? ☒ Yes ☐ No

Field work, collection and transport

Field conditions	tropical location with average temperature of 30 degrees celsius. Weather conditions were good during the two research cruises
Location	Chagos Archipelago region (-8 to -4°S; 70 to 73°E); Ile des Rats (71°18'35.8''E; 6°38'2''S); Manta Alley (71°21'19.8''E; 6°38'25.7''S). A detailed map of the bathymetry characteristics of the study sites is provided in Figure 1.
Access & import/export	2 days of ship steaming were needed to arrive in Egmont from the Maldives and 6 days from Seychelles (return in March 2020). No hard samples were collected for this study. Images only were collected. Deployed moorings were retrieved at the end of the cruise to avoid marine benthos littering. Research permits were provided by the British Indian Ocean Territory administration and the Foreign and Commonwealth Office.
Disturbance	The ROV did not touched the seabed as much as possible and stayed within the water column to avoid seabed damage

Reporting for specific materials, systems and methods

We require information from authors about some types of materials, experimental systems and methods used in many studies. Here, indicate whether each material, system or method listed is relevant to your study. If you are not sure if a list item applies to your research, read the appropriate section before selecting a response.

Materials & experimental systems

n/a	Involved in the study
☒	☐ Antibodies
☒	☐ Eukaryotic cell lines
☒	☐ Palaeontology and archaeology
☒	☐ Animals and other organisms
☒	☐ Clinical data
☒	☐ Dual use research of concern
☒	☐ Plants

Methods

n/a	Involved in the study
☒	☐ ChIP-seq
☒	☐ Flow cytometry
☒	☐ MRI-based neuroimaging
